# Evaluation of the *in vitro* Activity and *in vivo* Efficacy of Anidulafungin-Loaded Human Serum Albumin Nanoparticles Against *Candida albicans*

**DOI:** 10.3389/fmicb.2021.788442

**Published:** 2021-12-14

**Authors:** Yu Zhang, Yan-Chao Liu, Si-Min Chen, Hui Zong, Wei-Tong Hou, Xi-Ran Qiu, Shi-Yu Guo, Yu-Fang Sun, Yuan-Ying Jiang, Mao-Mao An, Hui Shen

**Affiliations:** ^1^Department of Pharmacology, Shanghai Tenth People’s Hospital, School of Medicine, Tongji University, Shanghai, China; ^2^Department of Clinical Pharmacy, Shanghai General Hospital, School of Medicine, Shanghai Jiao Tong University, Shanghai, China; ^3^Department of Clinical Laboratory Medicine, Shanghai Tenth People’s Hospital, School of Medicine, Tongji University, Shanghai, China

**Keywords:** anidulafungin, nanoparticles, *Candida albicans*, human serum albumin, antifungal

## Abstract

Recent decades have seen a significant increase in invasive fungal infections, resulting in unacceptably high mortality rates. Anidulafungin (AN) is the newest echinocandin and appears to have several advantages over existing antifungals. However, its poor water solubility and burdensome route of administration (i.e., repeated, long-term intravenous infusions) have limited its practical use. The objective of this study was to develop anidulafungin-loaded Human Serum Albumin (HSA) nanoparticles (NP) so as to increase both its solubility and antifungal efficacy. HSA was reduced using SDS and DTT, allowing liberation of free thiols to form the intermolecular disulfide network and nanoassembly. Reduced HSA was then added to MES buffer (0.1 M, pH 4.8) and magnetically stirred at 350 rpm and 25°C with AN (m/m 50:1) for 2 h to form nanoparticles (AN NP). We next performed routine antifungal susceptibility testing of *Candida* strains (*n* = 31) using Clinical and Laboratory Standards Institute (CLSI) methodologies. Finally, the *in vivo* efficacy of both AN and AN NP was investigated in a murine model of invasive infection by one of the most common fungal species—*C. albicans.* The results indicated that our carrier formulations successfully improved the water solubility of AN and encapsulated AN, with the latter having a particle size of 29 ± 1.5 nm with Polymer dispersity index (PDI) equaling 0.173 ± 0.039. *In vitro* AN NP testing revealed a stronger effect against *Candida* species (*n* = 31), with Minimum Inhibitory Concentration (MIC) values 4- to 32-fold lower than AN alone. In mice infected with *Candida* and having invasive candidiasis, we found that AN NP prolonged survival time (*P* < 0.005) and reduced fungal burden in kidneys compared to equivalent concentrations of free drug (*P* < 0.0001). In conclusion, the anidulafungin nanoparticles developed here have the potential to improve drug administration and therapeutic outcomes for individuals suffering from fungal diseases.

## Introduction

Invasive fungal infections remain a continuous and serious threat to human health. Globally, they are associated with approximately one and a half million deaths each year (Brown et al., 2012a). More specific estimates support a 30–40% mortality for invasive candidiasis, 20–30% for disseminated cryptococcosis, and a similar percentage for invasive aspergillosis (Campoy and Adrio, 2017). Such infections are very common in compromised individuals, including patients in medical or surgical intensive care units, patients with HIV infection/AIDS, and those undergoing solid organ transplantation or hematopoietic stem-cell transplantation (HSCT) (Denning, 1998; Vazquez and Sobel, 2006). Four classes of antifungal agents are clinically available—azoles, echinocandins, polyenes, and pyrimidine analogs; however, they collectively have had only modest success in reducing the high mortality rates of invasive mycoses like candidiasis and cryptococcosis (Brown et al., 2012b; Denning and Bromley, 2015).

Anidulafungin (AN) is a new echinocandin antifungal agent and—like other echinocandins—it selectively inhibits one target. For AN, this target is 1,3- beta-D-glucan synthesis, which is a major structural component of the fungal cell wall (Murdoch and Plosker, 2004). Results of current studies have indicated that AN appears to have several advantages over other antifungal drugs, including improved safety and efficacy (Vazquez and Sobel, 2006). However, AN has its own limitations, including its high, water insolubility. Moreover, it has a burdensome route of administration, as it is administered intravenously at a low concentration (∼0.8 mg/mL) over lengthy, repeated infusions (1.4 mL/min for 180 min on the 1st day, followed by daily administration for at least 14 days). Ultimately, these constraints have limited the practical use of this potent drug (Patel and Charneski, 2006). It is also worth noting that the high cost of echinocandins in comparison to azole antifungals may also attenuate their use as first-line agents in treating invasive fungal infections (Wilke, 2011).

Currently, colloidal drug carrier systems have been widely studied, owing to their major advantages of modifiable body distribution and enhanced cellular uptake (Kreuter, 1983; Schäfer et al., 1992). In addition, protein-based colloidal systems are particularly promising, since they are biodegradable, non-antigenic, relatively, easy to prepare, and have increased efficacy (Weber et al., 2000; Dreis et al., 2007). For instance, Vera-González et al. (2020) produced three different liposomal nanoparticles incorporating AN. Critically, those liposomes increased AN solubility and had promising efficacy (Vera-González et al., 2020). However, the liposome manufacturing has several limitations, including their high cost, quality assurance concerns, and issues regarding liposome stability during storage. Moreover, there are also difficulties regarding liposome sterilization. Collectively, these limitations have impeded their clinical translation (Qu et al., 2019).

As the most abundant plasma protein, human serum albumin (HSA) has emerged as a versatile drug delivery platform. This is due to several factors, including its good biocompatibility, non-toxicity, and non-immunogenic properties, as well as its biologically benign degradation file (Karimi et al., 2016). Numerous therapeutic drugs are delivered by HSA (Kratz, 2008). For example, the albumin paclitaxel (PTX) nanoparticle (Abraxane^®^) has already been approved by the FDA (Food and Drug Administration) for the treatment of metastatic breast cancer (Pinder and Ibrahim, 2006; Palumbo et al., 2016).

Here, we have developed anidulafungin-loaded HSA nanoparticles (AN NP) that address the aforementioned limitations and allow for enhanced antifungal therapy. This study presents an approach for the direct assembly of molecular HSA into larger-sized nanostructures with reconstructed, intermolecular disulfide bonds and hydrophobic interactions. The rich binding sites of AN within the HSA nanostructure enabled subsequent AN drug loading. In this fashion, AN NP were prepared and their antifungal efficacy was increased both *in vitro* and *in vivo*.

## Materials and Methods

### Ethics

All experimental procedures involving animals were performed in accordance with the Regulations for the Administration of Affairs Concerning Experimental Animals and approved by the State Council of the People’s Republic of China. The protocol used was approved by the Institutional Animal Care and Use Committee of Tongji University (Permit Number: TJAA08021101).

### Preparation of Anidulafungin NP

**FIGURE 1:**
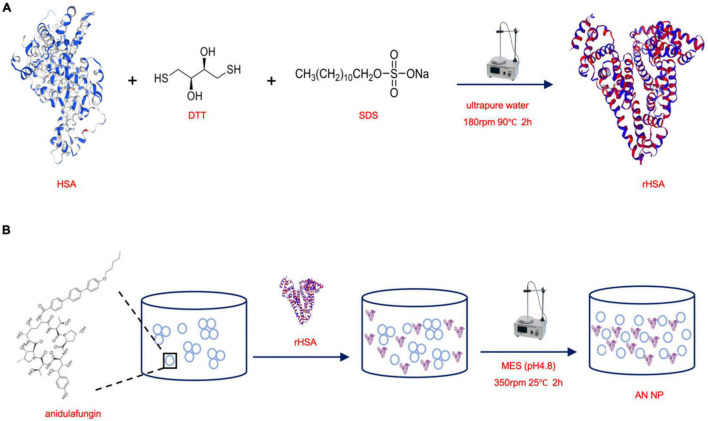
Scheme of synthesizing rHSA and AN NP. **(A)** HSA powder was dispersed in SDS and DTT, then the mixture was heated to 90°C and magnetically stirred at 180 rpm for 2 h to obtain reduced HSA (rHSA). **(B)** rHSA in MES buffer was magnetically stirred at 350 rpm and 25°C with AN (m/m 50:1) for 2 h to form the AN nanoparticles (AN NP).

HSA powder was dispersed in SDS (0.6%) and DTT (2.4 mM), resulting in a solution with a concentration of 10 mg/mL. According to our previously published method, the mixture was then heated to 90°C and magnetically stirred at 180 rpm for 2 h to obtain reduced HSA (rHSA) (Liu et al., 2020) (Figure 1A). Then, rHSA in MES buffer was magnetically stirred at 350 rpm and 25°C with AN (m/m 50:1) for 2 h to form the AN nanoparticles (AN NP) (Figure 1B). To prepare the HSA nanoparticles (HSA NP), rHSA in MES buffer was magnetically stirred at 350 rpm and 37°C for 4.5 h to form HSA NP. To characterize the nanoparticles, AN NP and HSA NP were dialyzed using commercially available 3KD dialysis bags.

### Cytotoxicity Test Using the LDH Release Assay and Live/Dead Cell Staining

RAW264.7 macrophages were used to evaluate the toxicity of HSA NP. Macrophages (1.25 × 10^5^ cells/mL) in DMEM containing 10% FBS were seeded in 96-well tissue culture plates and different concentrations of HSA NP (experimental) was added. Cytotoxicity was tested using LDH Cytotoxicity Assay Kit (Beyotime, Shanghai, China) and LIVE/DEAD Viability/Cytotoxicity Kit (Thermo Fisher, MA, United States) according to manufacturer‘s recommendations. lactate dehydrogenase (LDH) release was assessed by measuring absorbance at 490 and 600 nm and cellular viability (i.e., live/dead) was observed using the Automated Live Cell Imager (Lionheart FX, Biotek, Vermont, United States) using the GFP and PI channels.

### Characterization of Anidulafungin NP

Anidulafungin NP particle size was determined by dynamic light scattering (DLS) (Zetasizer Nano ZS, Malvern, Shanghai, China). The morphology was observed by transmission electron microscopy (TEM, Tecnai-12Bio-Twin, FEI, Netherlands). The effective encapsulation of AN into nanoparticles was confirmed by UV spectral analysis (Varian, Hong Kong, China). To evaluate stability, AN NP was incubated at either 4°C or room temperature for 30 days; alternatively, AN NP was also incubated at 37°C for 48 h. All measurements were conducted in triplicate. The solubility of 125 μg/mL AN with (experimental) or without (control) different ratios of rHSA in PBS was visually observed at 0 and 24 h. Two independent experiments were conducted.

### Fungal Strain and Cell Line

*Candida albicans* SC5314 was kindly provided by Sanglard D (Centre Hospitalier Universitaire Vaudois). *C. albicans* ATCC 90029 and clinical strains (379, 385, 395, 537, 538, 541, 0511311, 0710253, 0710419, 0710425, 0710448, 0710452, and 0710492), *C. tropicalis* standard strains ATCC 20026, ATCC 750, and clinical strains (98F, 267, 785, 909, and 1041), *C. glabrata* standard strains ATCC 28226, ATCC 1182, and clinical strains (337, 338), *C. parapsilosis* standard strains ATCC 22019 and clinical strains (642, 644), *C. krusei* standard strains ATCC 6258, *C. guilliermondii* standard strains ATCC 6260, *C. Auris* standard strains 1212 were from the fungi collection of our laboratory. All of other the other standard reference fungal strains (*n* = 8) were obtained from American Tissue Culture Collection (ATCC). All clinical fungal isolates (*n* = 23) were isolated from Shanghai Tenth People’s Hospital, Shanghai, China. All fungal strains were routinely cultured on sabouraud dextrose agar (SDA) plates (1% peptone, 4% dextrose, and 1.8% agar) for isolation of individual clones and cultured in yeast peptone dextrose (YPD) liquid medium (1% yeast extract, 2% peptone, and 2% dextrose) at 30°C in a shaking incubator.

RAW264.7 macrophages were obtained from the Cell Resources center of the Chinese Academy of Sciences and were cultured in DMEM medium containing 10% fetal bovine serum (FBS) at 37°C and 5% CO_2_.

### *In vitro* Antifungal Susceptibility Testing

To determine *in vitro* antifungal activity, a broth microdilution susceptibility assay was conducted in RPMI 1640 medium in accordance with the Clinical and Laboratory Standards Institute (CLSI) guidelines. Briefly, strains in RPMI 1640 medium (approximate final concentration = 1 × 10^3^ CFU/mL) were prepared in 96-well plates; after preparation, antifungal agents (100 μg/mL) were selectively added. The final concentrations ranged from 0.002 to 1 μg/mL for AN and AN NP. HSA NP with the same mass and addition volume as AN NP was used as control to explore their antifungal efficacy against *C. albicans* SC5314. The plates were then incubated at 35°C for 24 h. MIC results were determined after incubation and were made according to inhibition of visible growth when compared to that of control. The MIC range of fluconazole to *C. parapsilosis* ATCC 22019 from 0.5 to 1 μg/mL, indicating the test was acceptable. Two independent experiments were conducted and the average used for later analysis.

### Time-Kill Kinetics Assay

Strains were grown in YEPD medium with a starting inoculum of 5 × 10^5^ CFU/mL. AN and AN NP (0.25 μg/mL for *Candida albicans* SC5314, 0.5 μg/mL for *Candida albicans* 538) and PBS (control) were tested for *Candida albicans*. After incubation with agitation at 30°C and at predetermined time points, 100 μL aliquots were obtained from each solution and serially diluted in PBS. A 100 μL aliquot from each dilution was then spread on a Sabouraud dextrose agar (SDA) plate. Colony counts were determined after incubation at 35°C for 48 h. Each group coated three plates at each predetermined time points. Two independent experiments were conducted and the average used for later analysis.

### Paper Disk Agar Diffusion Assay

To examine the effect of AN and AN NP on the growth of different *Candida* species, we performed a paper disk-diffusion assay in agar. Briefly, a cell suspension of either *Candida albicans* SC5314 or 538 in PBS (5 mL, 1 × 10^5^ CFU/mL) was poured evenly over a pre-prepared SDA plate. The plate was allowed to stand for 30 min, after which the supernatant was removed. The plate was then allowed to dry for an additional 30 min. After the top layer had solidified, sterile paper disck (8 mm) were placed on the agar surface and test agents were added. Five types of inhibition zones were noted: (a) 1 μg for AN; (b) AN NP containing 1 μg for AN; (c) 2 μg for AN; (d) AN NP containing 2 μg for AN; (e) PBS. After incubating at 35°C for 24 h, the size and pattern of the growth inhibition zone around the disks on the agar were evaluated. Two independent experiments were conducted and the average used for later analysis.

### Animal Model

*Candida* yeast was selected to use during the *in vivo* assays because it is one of the most common fungal agents that causes life-threatening invasive fungal infections in humans. Of the strains implicated, *C. albicans* is the most prevalent (Brown et al., 2012a). An established murine model of invasive candidiasis with some modifications was used to evaluate the *in vivo* efficacy of AN and AN NP. Immunocompetent 7–8-week-old female C57BL/6 mice weighing 18–20 g were used in this study. The mice were randomly divided into groups according to their body weight, and then the random numbers were generated by Excel for simple random grouping. To establish invasive candidiasis, mice were injected with 1 × 10^6^ CFU of *C. albicans* SC5314 intravenously *via* the lateral tail vein.

### Survival

In the survival study, antifungal treatments began 2 h after fungal inoculation as a single dose. Different treatment groups (*n* = 8 mice per group) were as follows: Vehicle control (PBS), 0.3 mg/kg of AN or AN NP (containing 0.3 mg/kg AN). AN and AN NP were diluted in PBS and intravenously administered *via* the lateral tail vein in a volume of 0.3 mL. Post-infection, mice were monitored and recorded regarding their survival twice daily for a total of 6 days. Two independent experiments were conducted.

### Fungal Burden and Histopathology

To assess fungal burden, antifungal drug was administered once began 2 h after fungal inoculation and continued for 2 days. Two days after administration, mice were humanely euthanized and kidneys were collected for quantitative determination of tissue fungal burden (*n* = 5 mice per group) and histological staining (*n* = 3 mice per group). After kidney weights were determined, tissues were homogenized in 1 mL PBS. Homogenates were serially diluted in 10-fold steps, and aliquots (100 μL) of the resulting homogenates were plated on SDA plates. The plates were incubated at 35°C for 48 h, after which the number of CFU were counted. All fungal burdens are indicated as CFU/g. All SDA plates were performed in duplicate and the average used for later analysis. For histopathology, the kidneys were fixed in 10% neutral formalin for HE and PAS staining.

### Statistical Analysis

Time-kill kinetics assay data and fungal burden data were analyzed using one-way analysis of variance (ANOVA), and survival curves were compared using the Log-rank (Mantel–Cox) test. A *P*- value of ≤0.05 was considered statistically significant. Software Graphpad prism 7 was used to evaluate data.

## Results

### Carrier Human Serum Albumin NP Was Safe and Non-toxic in RAW264.7 Macrophages

**FIGURE 2:**
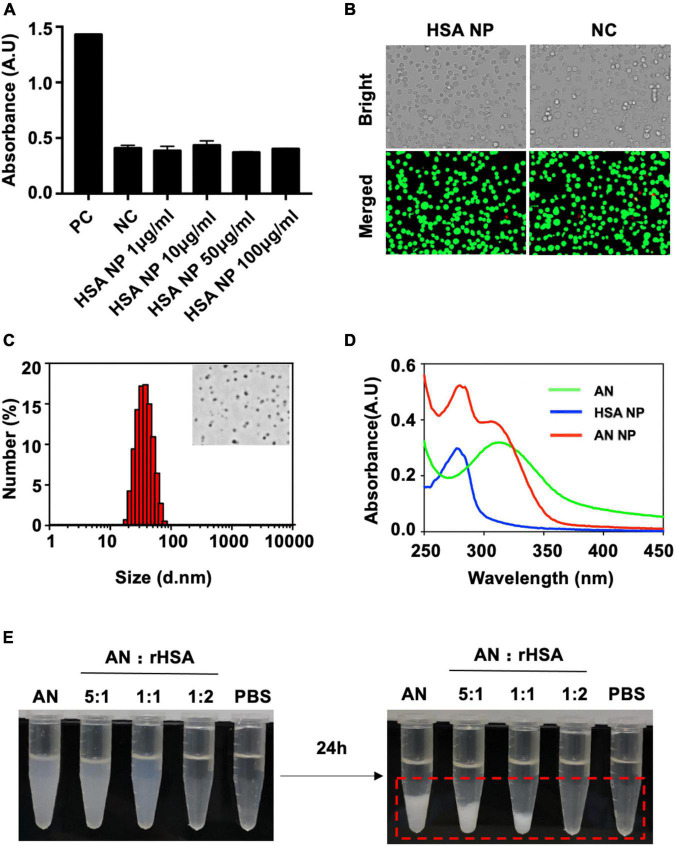
Cytotoxicity, characterization, and dispersibility analysis of produced nanoparticles. **(A)** RAW264.7 macrophages incubated overnight were separated into the following groups: Positive control (PC), negative control (NC), or experimental groups receiving different concentrations of HSA NP for 3 h. LDH was then released by cells, detected, and measured. **(B)** Cellular viability staining (i.e., live/dead assessment of cells) of RAW264.7 macrophages in either the NC (0 μg/mL, 4 h) or HSA NP (50 μg/mL, 4 h) groups. Red indicates dead cells and green indicates live cells. **(C)** Hydrodynamic size of AN-NP as determined by DLS. The inset figure is a representative TEM image of AN-NP. **(D)** UV-vis absorbance spectra of AN, HSA NP, and AN NP. **(E)** Dispersibility of mixture of rHSA and AN (250 μg/mL) with an indicated ratio in PBS buffer at room temperature for 24 h. Red dotted box indicates visible coagulation.

In this work, we reduced HSA using SDS and DTT to liberate free thiols and allow for the formation of an intermolecular disulfide network. As a result, a nanoassembly was formed, resulting in the obtained HSA NP. Given this, we first sough to determine the safety of our HSA NP. The cytotoxicity of HSA NP was evaluated using a LDH release assay along with cellular viability staining (i.e., determining live/dead cells) in RAW264.7 macrophages. The quantity of LDH released by different concentrations of applied HSA NP was the same as that observed in the negative control. The positive control was approximately of three times that of either HSA NP or NC (Figure 2A). The cellular viability of cells incubated with either HSA NP or NC is shown in Figure 2B. As indicated, live cells are indicated in green and dead cells in red. Importantly, the ratio of living to dead cells across these two groups was consistent and not statistically different from each other.

### Characteristics of Anidulafungin NP

Figure 2C shows the particle size and representative TEM image of AN NP. AN NP particle size was determined and expressed as mean particle size (in nanometers) ± standard deviation (SD). The particle size was 29 ± 1.5 nm with PDI of 0.173 ± 0.039. TEM imaging revealed that AN NP exhibited uniform and spherical morphology with a diameter of approximately 30–50 nm.

The aggregation state of AN in rHSA (AN NP) was evaluated by measuring UV-visible absorbance (Figure 2D). AN and HSA NP was used as contrast. The absorption spectra of AN and HSA NP showed separate, highly pronounced peaks at 310 and 277 nm, respectively. The absorption spectrum of AN NP showed two highly pronounced peaks at 280 and 306 nm.

**FIGURE 3:**
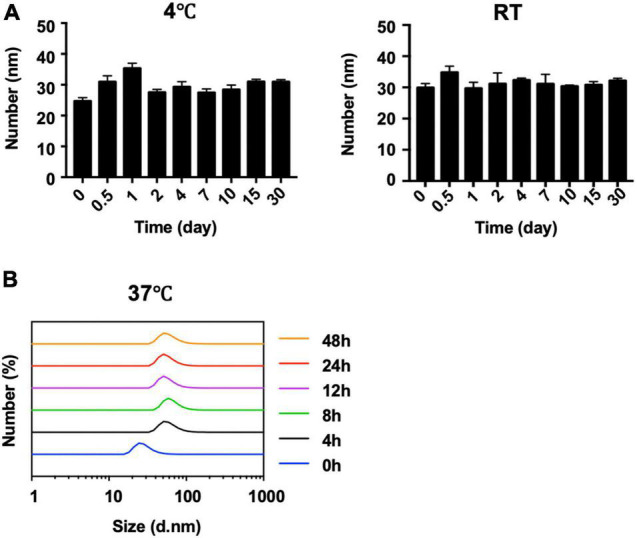
Stability of AN NP. **(A,B)** Long-term stability analysis of AN NP at 4°C and room temperature (RT) **(A)** for 30 days, and 37°C for 48 h **(B)** by size change determination.

The behavior of AN with or without different ratios of rHSA was measured in PBS at room temperature (Figure 2E). When the ratio of rHSA and AN was 0, 1:5, 1:1, or 2:1, the mixed liquid went from turbid to clear at 0 h and a white precipitate gradually formed in less than 24 h. PBS was used as the negative control. In addition, long-term stability analysis indicated that the size distribution of AN-NP did not change markedly within 30 days—either at 4°C or room temperature (Figure 3A) or at 37°C within 48 h (Figure 3B). Considering that poor dispersibility has limited the *in vitro* and *in vivo* antifungal efficacies of AN, we hypothesized that the improved solubility and dispersibility of AN-NP would predict its superior antifungal activity compared to AN.

### Anidulafungin NP Exhibited Stronger *in vitro* Antifungal Efficacy Against *Candida* Species Than Anidulafungin

Anidulafungin had low solubility and poor dispersibility in aqueous solution (Patel and Charneski, 2006). Our results indicated that nanoassembly with HSA markedly improved the dispersibility of AN, making it possible to enhance its antifungal activity (Figure 2E). Given this, we first compared the MIC of AN and AN-NP against the common pathogenic *Candida* spp., including 9 standard reference strains and 23 clinical isolates. This was done using a broth microdilution susceptibility assay according to CLSI guidelines M27-A3. First of all, we determined the value of HSA NP was the same as that in the non-dosing group. Next our results showed that AN and AN NP were active against all 32 strains tested, with MIC values ranging from 0.125 to 2 mg/L and 0.0313 to 0.5 mg/L, respectively (Table 1). Obviously, the efficacy of AN NP against *Candida* strains was found to be superior to AN, with the exception of the *C. tropicalis* standard strain ATCC 20026. AN NP exhibited a stronger effect against *Candida* species (8 standard reference strains and 23 clinical isolates), with MIC values 4- to 32-fold lower than those of AN.

**TABLE 1:** MICs of AN and AN NP against common pathogenic fungi.

Yeast strains	AN (mg/L)	AN NP (mg/L)
**Standard strains**		
*C. albicans* SC5314	0.5	0.0313
*C. albicans* ATCC 90029	1	0.0625
*C. tropicalis* ATCC 20026	0.125	0.0625
*C. tropicalis* ATCC 750	1	0.0313
*C. glabrata* ATCC 28226	0.5	0.0625
*C. glabrata* ATCC 1182	0.5	0.0625
*C. parapsilosis* ATCC 22019	1	0.125
*C. krusei* ATCC 6258	0.5	0.0625
*C. guilliermondii* ATCC 6260	0.5	0.125
**Clinical strains**		
*C. albicans* 379	0.5	0.0625
*C. albicans* 385	0.5	0.0625
*C. albicans* 395	0.5	0.0625
*C. albicans* 537	0.125	0.0313
*C. albicans* 538	0.5	0.0313
*C. albicans* 541	0.25	0.0625
*C. albicans* 0511311	0.5	0.0625
*C. albicans* 0710253	0.5	0.0625
*C. albicans* 0710419	0.5	0.0625
*C. albicans* 0710425	0.5	0.0625
*C. albicans* 0710448	1	0.0625
*C. albicans* 0710452	0.5	0.0625
*C. albicans* 0710492	1	0.0625
*C. tropicalis* 98F	0.5	0.0313
*C. tropicalis* 267	0.5	0.0625
*C. tropicalis* 785	0.25	0.0625
*C. tropicalis* 909	1	0.125
*C. tropicalis* 1041	0.5	0.0313
*C. glabrata* 337	1	0.0313
*C. glabrata* 338	1	0.0625
*C. parapsilosis* 642	2	0.25
*C. parapsilosis* 644	2	0.5
*C. Auris* 1212	2	0.0625

**FIGURE 4:**
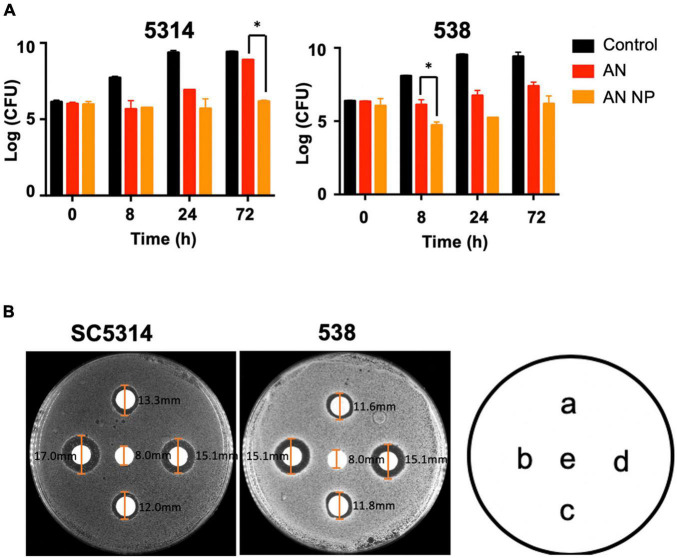
AN NP exhibits stronger activity against *Candida albicans* compared to AN. **(A)** Time-kill kinetics assay of *Candida albicans* SC5314 and 538 treated with AN or AN NP. Strains were grown in YEPD medium with a starting inoculum of 5 × 10^5^CFU/mL. *P < 0.05 (one-way analysis of variance). **(B)** Antifungal susceptibility testing of *Candida albicans* SC5314 and 538 using 8 mm disks. a, c represents, respectively 1 μg and 2 μg AN, respectively; b, d represents AN NP (containing 1 μg or 2 μg AN, respectively); e represents PBS. The size of each inhibition zone diameter is marked on the figure.

Furthermore, we investigated the *in vitro* drug susceptibility of AN and AN NP using a time-kill kinetics assay along with a paper disk agar diffusion test. The time-kill kinetics assay was conducted against *C. albicans* SC5314 and 538. As shown in Figure 4A and when compared with control, AN and AN NP significantly inhibited the growth of *C. albicans* SC5314 and 538. AN NP showed a stronger inhibitory effect relative to AN and exhibited fungistatic effect against both strains.

The paper disk ager diffusion test revealed that AN and AN NP significantly inhibited the growth of *C. albicans* SC5314 and 538 when compared with PBS (Figure 4B). Compared with AN (1 μg or 2 μg), AN NP containing either 1 μg or 2 μg of AN revealed a dose-independent increase in zone diameter. Meanwhile, AN and AN NP exhibited fungistatic effects against both strains.

### Anidulafungin NP Prolonged the Survival Time and Reduced Fungal Burden in Kidneys Compared to Anidulafungin in Mice With Invasive Candidiasis

**FIGURE 5:**
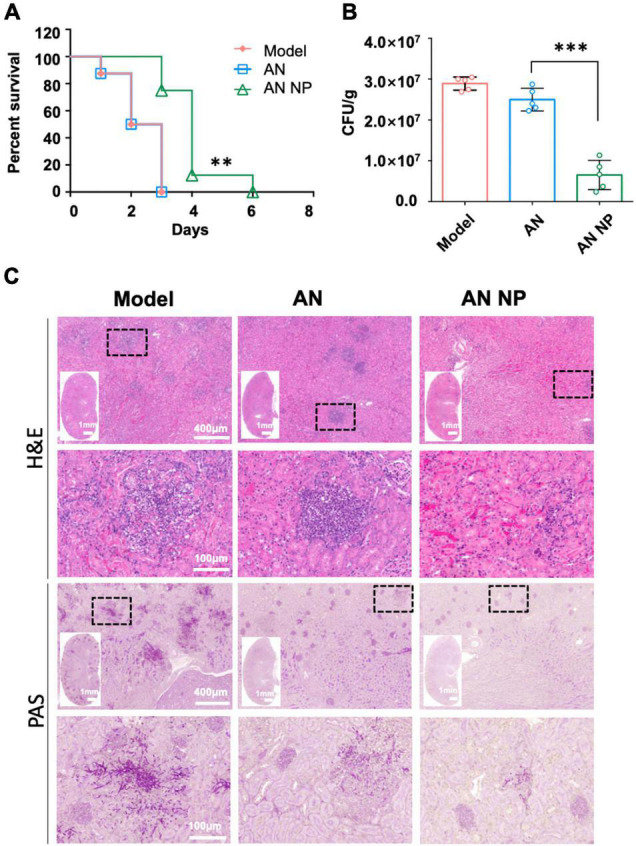
AN NP is much more effective than AN for treating invasive candidiasis caused by *C. albicans*. **(A)** Survival curves for mice with *Candida albicans* treated with antifungal agents. Mice were monitored without therapy until day 6. **P < 0.005 versus the results for the control [Log-rank (Mantel–Cox) test]. **(B)** Fungal burdens in mice with invasive *Candida albicans* treated with antifungal agents. Mice were humanely euthanized 2 days after treatment; kidney samples were then collected for fungal burden analysis. Horizontal lines represent mean values, and whiskers representing standard deviations. ***P < 0.0001 versus the result for the control (one-way analysis of variance). **(C)** Representative H&E and PAS staining of kidneys from *C. albicans* SC5314-infected mice with indicated treatment at day two, post-infection. Black dotted frame indicates inflammatory cells (H&E) and hypha (PAS) in the tissues.

In the 6-day survival experiment, AN NP exhibited potent efficacy in our *in vivo*, invasive candidiasis model. We assessed the *in vivo* effects of AN NP at a dose of 0.3 mg/kg, with AN and PBS as controls (Figure 5A). The result showed that 0.3 mg/kg of AN NP protected the mice through the observation period and somewhat improved their overall survival rate. All mice in the control groups were dead at 3 days post-inoculation, with a median survival time of 2.5 days. Compared with the survival time for the control groups, AN NP significantly prolonged the survival time in mice (*P* < 0.005). The median survival time of mice in the AN NP group was 4 days.

To further examine the protective role of AN NP in host defense against *C. albicans* infection, we surveyed the fungal burden and histopathology of the kidneys, which are the organs most vulnerable to *C. albicans* infection. For this assessment, AN and PBS were used as controls. Following 2 days of antifungal treatment, we examined changes in the fungal burden in the kidneys. As indicated by the results shown in Figure 5B, a significant reduction in fungal burden was observed after AN NP treatment. The CFU counts for the AN NP-treated group (0.3 mg/kg) were significantly lower than those of the AN group. More specifically, the mean value for the AN NP group was 0.6 × 10^7^ CFU/g while the mean value for the AN group was 2.5 × 10^7^ CFU/g (*P* < 0.0001). Histopathological studies of renal tissue of all mice treated with AN NP at 0.25 mg/kg showed improved infection status compared to the control groups (Figure 5C). Signs of improved infection status in the renal tissue of mice treated with AN NP included decreased areas with inflammatory cells and reduced numbers of fungal hyphae (surrounding tubules and glomeruli).

## Discussion

In this work, we have presented the first study showing the encapsulation of AN in HSA to form therapeutic nanoparticles. Inspiration for the AN NP was the approval of Abraxane—the characteristic example of albumin-based NPs—in 2005 by the United States Food and Drug Administration (FDA) (Yardley, 2013). It was developed to surmount the low water solubility problem of the anticancer agent paclitaxel (Jahanban-Esfahlan et al., 2019). Similarly, the highly water insoluble nature of AN has limited the practical use of this potent drug (Patel and Charneski, 2006; Kristina et al., 2009). Critically, our nanoparticles successfully showed a way around this problem. In our study, increasing rHSA content resulted in improved water solubility of AN (Figure 2E). Importantly, this approach allowed for greatly enhanced *in vitro* and *in vivo* antifungal efficacy of AN. In addition, we propose the improvement of drug efficacy may shorten the course of treatment and reduce the dose to reduce adverse reactions.

Human serum albumin was reduced by DTT and SDS into larger-sized nanostructures with reconstructed intermolecular disulfide bonds and hydrophobic interactions. The rich binding sites of AN within the HSA nanostructures enabled AN loading. As shown in Figure 2D, the UV-visible analysis of AN NP showed an obvious, characteristic AN peak at approximately 306 nm, indicating successful incorporation of AN into the rHSA-formed nanostructures.

Prior to investigating the antifungal activity of our nanoparticles, we first examined whether these nanoparticles were safe. The cytotoxicity test of HSA NP in RAW264.7 macrophages demonstrated the DTT and SDS used to reduce HSA was effectively removed. Critically, our results showed both *in vitro* and *in vivo* safety of AN NP.

*Candida* yeast was selected because it is one of the most common fungal agents that causes life-threatening invasive fungal infections in humans. Of the strains implicated, *C. albicans* is the most prevalent (Brown et al., 2012a). Given this, we first investigated the *in vitro* susceptibility to AN NP and AN of several strains, including *Candida albicans*, *Candida krusei*, *Candida tropicalis*, *Candida glabrata*, *Candida parapsilosis*, *Candida guilliermondii*, and *Candida Auris*. The results showed that both AN and AN NP were active against all 32 strains tested. As expected, the efficacy of AN NP against *Candida* strains was superior to that of AN, with MIC values 4- to 32-fold lower than AN (except for *C. tropicalis* ATCC 20026). Standard strain SC5314 and clinical strain 538 of *Candida albicans* were further used to investigate the *in vitro* drug susceptibility of AN and AN NP using both a time-kill kinetics assay and paper disk agar diffusion test. Results of both experiments demonstrated that AN NP exhibited stronger antifungal effects than AN.

Furthermore, the results of our survival and fungal burden experiments demonstrated that AN NP also had better efficacy than AN in the murine model of invasive candidiasis caused by *C. albicans*. Compared with the survival time for the control groups—which had been administrated AN—AN NP significantly prolonged the survival time in mice (*P* < 0.005) at a dose of 0.3 mg/kg. When assessing the effects on kidney fungal burdens, AN NP significantly decreased fungal burden when compared with AN (*P* < 0.0001). Moreover, our renal histopathological study showed the existence of renal damage (Figure 5) when mice were treated intravenously (i.v.) with PBS. When renal damage was compared between AN and AN NP, it was observed that the presence of AN NP alleviated renal damage. Therefore, we concluded that AN NP had a notably stronger protective effect than AN in mice with *C. albicans* infection.

In summary, we have developed and characterized a novel antifungal formulation. More importantly, this formulation has been shown to have a stronger *in vitro* efficacy against *Candida* strains and *in vivo* protective potential in a murine model of *C. albicans* infection than AN. This improved antifungal efficacy will allow AN NP to be a viable alternative to AN, considering that the frequency of administration and treatment cost would both be greatly decreased. Further study will be needed to better understand the use and applicability of AN NP.

## Data Availability Statement

The original contributions presented in the study are included in the article/supplementary material, further inquiries can be directed to the corresponding authors.

## Ethics Statement

The animal study was reviewed and approved by all experimental procedures involving animals were performed in accordance with the Regulations for the Administration of Affairs Concerning Experimental Animals and Approved by the State Council of the People’s Republic of China. The protocol used was approved by the Institutional Animal Care and Use Committee of Tongji University (Permit Number: TJAA08021101).

## Author Contributions

YZ: experimental designer and executor of this research, completing data analysis, and writing and revising the first draft of the manuscript. Y-CL: conceptualization, methodology, and investigation. S-MC: conceptualization, methodology, and writing – review and editing. HZ, W-TH, X-RQ, S-YG, and Y-FS: resources, validation, investigation, and visualization. Y-YJ, M-MA, and HS: conceptualization, supervision, project administration, and writing – review and editing. All authors contributed to the article and approved the submitted version.

## Conflict of Interest

The authors declare that the research was conducted in the absence of any commercial or financial relationships that could be construed as a potential conflict of interest.

## Publisher’s Note

All claims expressed in this article are solely those of the authors and do not necessarily represent those of their affiliated organizations, or those of the publisher, the editors and the reviewers. Any product that may be evaluated in this article, or claim that may be made by its manufacturer, is not guaranteed or endorsed by the publisher.

## References

[B1] Brown G. D. Denning D. W. Gow N. A. Levitz S. M. Netea M. G. White T. C. (2012a). Hidden killers: human fungal infections. *Sci. Transl. Med.* 4:4404. 10.1126/scitranslmed.3004404 23253612

[B2] Brown G. D. Denning D. W. Levitz S. M. (2012b). Tackling human fungal infections. *Science* 336:647. 10.1126/science.1222236 22582229

[B3] Campoy S. Adrio J. L. (2017). Antifungals. *Biochem. Pharmacol*. 133 86–96. 10.1016/j.bcp.2016.11.019 27884742

[B4] Denning D. W. (1998). Invasive aspergillosis. *Clin. Infect Dis.* 26 781–803. 10.1086/513943 9564455

[B5] Denning D. W. Bromley M. J. (2015). Infectious disease. how to bolster the antifungal pipeline. *Science* 347 1414–1416. 10.1126/science.aaa6097 25814567

[B6] Dreis S. Rothweiler F. Michaelis M. Cinatl J. J. Kreuter J. Langer K. (2007). Preparation, characterisation and maintenance of drug efficacy of doxorubicin-Loaded human serum albumin (HSA) nanoparticles. *Int. J. Pharm.* 341 207–214. 10.1016/j.ijpharm.2007.03.036 17478065

[B7] Jahanban-Esfahlan A. Ostadrahimi A. Jahanban-Esfahlan R. Roufegarinejad L. Tabibiazar M. Amarowicz R. (2019). Recent developments in the detection of Bovine serum albumin. *Int. J. Biol. Macromol.* 138 602–617. 10.1016/j.ijbiomac.2019.07.096 31319084

[B8] Karimi M. Bahrami S. Ravari S. B. Zangabad P. S. Mirshekari H. Bozorgomid M. (2016). Albumin nanostructures as advanced drug delivery Systems. *Expert Opin. Drug Deliv.* 13 1609–1623. 10.1080/17425247.2016.1193149 27216915 PMC5063715

[B9] Kratz F. (2008). Albumin as a drug carrier: design of prodrugs, drug conjugates and Nanoparticles. *J. Control Release* 132 171–183. 10.1016/j.jconrel.2008.05.010 18582981

[B10] Kreuter J. (1983). Evaluation of nanoparticles as drug-delivery systems. II: Comparison of the body distribution of nanoparticles with the body distribution of Microspheres (diameter greater than 1 micron), liposomes, and emulsions. *Pharm. Acta Helv.* 58 217–226. 6622500

[B11] Kristina E. E. Scott R. P. Karim A. C. Thomas J. W. (2009). Pharmacology and antifungal properties of anidulafungin, a new echinocandin. *Pharmacotherapy* 29 17–30. 19113794 10.1592/phco.29.1.17

[B12] Liu Y. Han Y. Fang T. Chen S.-M. Hu X. Song L. (2020). Turning Weakness into strength: Albumin nanoparticle-redirected amphotericin B Biodistribution for reducing nephrotoxicity and enhancing antifungal activity. *J. Control Release.* 324 657–668. 10.1016/j.jconrel.2020.05.026 32446873

[B13] Murdoch D. Plosker G. L. (2004). Anidulafungin. *Drugs* 64 2249–2258. 10.2165/00003495-200464190-00011 15456342

[B14] Palumbo R. Sottotetti F. Bernardo A. (2016). Targeted chemotherapy with Nanoparticle albumin-bound paclitaxel (nab-paclitaxel) in metastatic breast cancer: Which benefit for which patients? *Ther. Adv. Med. Oncol.* 8 209–229. 10.1177/1758834016639873 27239239 PMC4872255

[B15] Patel P. N. Charneski L. (2006). Anidulafungin (Eraxis) for Candida infections. *Med. Lett. Drugs Ther.* 48 43–44. 10.1097/01.aog.0000239499.97978.bc 16770294

[B16] Pinder M. C. Ibrahim N. K. (2006). Nanoparticle albumin-bound paclitaxel for Treatment of metastatic breast cancer. *Drugs Today* 42 599–604. 10.1358/dot.2006.42.9.1009902 17028669

[B17] Qu N. Sun Y. Li Y. Hao F. Qiu P. Teng L. (2019). Docetaxel-loaded human Serum albumin (HSA) nanoparticles: synthesis, characterization, and evaluation. *Biomed. Eng. Online* 18:11. 10.1186/s12938-019-0624-7 30704488 PMC6357434

[B18] Schäfer V. Briesen H. V. Andreesen R. Steffan A. M. Royer C. Tröster S. (1992). Phagocytosis of nanoparticles by human immunodeficiency virus (hlv)-infected macrophages: a possibility for antiviral drug targeting. *Pharm. Res.* 9 541–546. 10.1023/a:1015852732512 1495900

[B19] Vazquez J. A. Sobel J. D. (2006). Anidulafungin: a novel echinocandin. *Clin. Infect. Dis*. 43 215–222. 10.1086/505204 16779750

[B20] Vera-González N. Bailey-Hytholt C. M. Langlois L. Camargo Ribeiro F. Souza Santos E. L. Campos Junqueira J. (2020). Anidulafungin liposome Nanoparticles exhibit antifungal activity against planktonic and biofilm Candida Albicans. *J. Biomed. Mater Res. A* 108 2263–2276. 10.1002/jbm.a.36984 32363762

[B21] Weber C. Kreuter J. Langer K. (2000). Desolvation process and surface Characteristics of HSA-nanoparticles. *Int. J. Pharm.* 196 197–200. 10.1016/s0378-5173(99)00420-2 10699717

[B22] Wilke M. (2011). Treatment and prophylaxis of invasive candidiasis with Anidulafungin, caspofungin and micafungin and its impact on use and costs - review of the literature. *Eur. J. Med. Res.* 16 180–186. 10.1186/2047-783x-16-4-180 21486732 PMC3352074

[B23] Yardley D. A. (2013). nab-Paclitaxel mechanisms of action and delivery. *J. Control Release* 170 365–372. 10.1016/j.jconrel.2013.05.041 23770008

